# Functional limitations and loneliness in middle-aged and older adults: differentiating emotional loneliness and social loneliness

**DOI:** 10.1186/s12889-026-27997-8

**Published:** 2026-06-01

**Authors:** Johannes Beller, Julia Graßhoff, Stefanie Sperlich

**Affiliations:** 1 Medical Sociology, Medical University Lausitz – Carl Thiem, Thiemstraße 111, Cottbus, 03048 Germany; 2 Hannover Medical School, Hannover, Germany

**Keywords:** Functional limitations, Disability, Loneliness, Aging

## Abstract

**Background:**

People with functional limitations may be lonelier than those without functional limitations, as health-related constraints can restrict social participation. However, this relationship might also vary depending on the type of loneliness considered and the age of the individual. Consequently, this study examines differences in emotional and social loneliness according to functional limitation status and across age groups.

**Methods:**

Population-based data from 3,984 participants aged 40 and older from the 2023 German Aging Survey were analyzed. Emotional and social loneliness were measured using the De Jong Gierveld Loneliness Scale, and functional limitations were assessed using the Global Activity Limitation Indicator (GALI). Analysis of variance examined differences across age groups and functional limitation severity.

**Results:**

Results showed that participants with functional limitations generally reported progressively higher levels of both types of loneliness compared to those without functional limitations. Emotional loneliness showed consistent patterns across age groups, with progressive increases from no functional limitations to moderate functional limitations to severe functional limitations. In contrast, social loneliness displayed a significant interaction with age group, where middle-aged adults (40–65 years) showed much stronger progressions in social loneliness according to functional limitation severity as compared to older adults (66+).

**Conclusions:**

These findings suggest that functional limitations are generally associated with increased loneliness, but that the extent varies by both type of loneliness and age group. The stronger differences in social loneliness among middle-aged adults indicate that functional limitations may have particularly pronounced social implications earlier in the life course. Middle-aged adults with functional limitations thus represent a particularly psychosocially vulnerable group.

**Supplementary Information:**

The online version contains supplementary material available at 10.1186/s12889-026-27997-8.

## Background

Loneliness can be defined as the subjective perception of inadequate social relationships, reflecting a discrepancy between desired and actual social connections [1]. Two distinct components of loneliness can be distinguished: Emotional loneliness, which results from the perception of inadequate deep intimate relationships such as those with a romantic partner or best friend, and social loneliness, which results from the perception of inadequate broader group connections such as community ties [2]. Loneliness as a subjective feeling thus differs from social isolation, which represents the objective absence of social contacts and relationships [3]. While brief feelings of loneliness are common, persistent and severe loneliness poses significant risks to physical and mental health across the lifespan [4, 5]. The adverse health consequences of loneliness are extensive, encompassing deteriorated physical health, cognitive decline, and increased psychological distress [3, 6, 7]. These effects are mediated through multiple pathways, including compromised health behaviours, disrupted sleep patterns, and dysregulation of stress response systems such as the hypothalamic-pituitary-adrenal axis, which contributes to chronic inflammation and weakened immune function [8]. Moreover, chronic loneliness has been identified as a robust predictor of premature mortality, with effect sizes comparable to established risk factors such as smoking and obesity [4, 6].

Research also demonstrates pronounced age-related differences in loneliness. Loneliness follows a U-shaped distribution across age groups, with heightened prevalence among older adults compared to younger and middle-aged populations [9, 10]. The manifestation of loneliness also differs between age groups [11, 12]: Middle-aged adults typically experience loneliness related to work-life balance challenges, family caregiving responsibilities, and social network transitions as friendships become more difficult to maintain. In contrast, older adults may face loneliness primarily through cumulative losses including bereavement and declining health. Longitudinal evidence further supports these age-related patterns: Using a cross-lagged panel model, Abaei and Martin demonstrated that aging is associated with increases in loneliness and that there is a dynamic, reciprocal relationship between cognitive function and loneliness in late life [13, 14]. As such, studies on loneliness should be mindful of potential age differences. In the present study, we distinguish between middle-aged (40–65 years) and older adults (66 + years), reflecting a well-established categorization in life-span developmental research that captures the transition from working life to post-retirement phases with their distinct social contexts and role expectations. As reported below, sensitivity analyses confirmed that our findings are robust to a more differentiated three-group categorization.

People with functional limitations may be particularly vulnerable to experiencing loneliness [15]. Impairments can limit access to social environments, reduce opportunities for interpersonal connections, and create challenges in maintaining existing relationships. Additionally, societal stigma and discrimination toward individuals with functional limitations can further exacerbate feelings of loneliness. However, the relationship between functional limitations and loneliness might be complicated by the well-documented disability paradox, which suggests that individuals with significant functional limitations often report quality of life that is surprisingly similar to, or sometimes even higher than, those without functional limitations [16]. It is suggested that people with functional limitations are often able to develop effective coping strategies and alternative sources of support that buffer against the potentially isolating effects of their functional limitations [17]. Because the paradox has primarily been documented for global evaluations of quality of life and subjective well-being, it remains unclear whether the same buffering extends to specific psychosocial outcomes such as loneliness.

Thus, despite the seemingly established relationship between functional limitations and loneliness, significant gaps remain in our understanding of how loneliness varies with functional limitation severity and across age groups. For example, a recent systematic review by Gomez-Zuniga et al. highlighted that existing research has not adequately determined whether loneliness in the context of disability represents a single construct or should be differentiated into distinct subtypes, emphasizing the need for further investigation into different types of loneliness and their relationship to functional limitations [15]. While important empirical research such as Bishop et al. has provided valuable insights into persistent inequalities in regards to loneliness, this study measured loneliness as a unidimensional construct using a single item, which may not capture the multifaceted nature of loneliness in people with and without functional limitations [18]. The distinction between emotional loneliness, characterized by the perceived inadequacy of close intimate relationships, and social loneliness, characterized by the perceived inadequacy of broader social networks, may be particularly relevant for understanding the experiences of people with functional limitations. Furthermore, the impact of functional limitations on different types of loneliness may also vary across age groups, as middle-aged and older adults face distinct social challenges and have different social expectations and opportunities. However, studies are lacking that investigate differences in social and emotional loneliness according to functional limitation status. The current study strives to help close this gap in the literature. We ask: “How do people with functional limitations differ from those without functional limitations in their experience of emotional and social loneliness, and do these differences vary across age groups?” Based on prior research, we formulate the following hypotheses: (H1) Individuals with functional limitations report higher levels of both emotional and social loneliness compared to those without functional limitations, with a dose-response pattern such that greater limitation severity is associated with greater loneliness. (H2) The association between functional limitations and loneliness varies by age group, with middle-aged adults showing stronger differences in loneliness according to functional limitation status compared to older adults, particularly for social loneliness, given the greater social role demands and expectations characteristic of midlife.

## Methods

### Sample

The data used in this study were taken from the public release of the German Aging Survey (DEAS), a population-based study on Germans aged 40 and older that is conducted by the Research Data Center of the German Center of Gerontology [19]. The participants in the DEAS are recruited through probability sampling and previous participants are re-contacted for follow-up interviews. These interviews are conducted in-person at the participant’s residence and adhere to German law and the ethical standards of the 1964 Helsinki declaration. The 2023 wave of the DEAS was used for this research, which is the most recent wave available. A total of 3,984 participants in 2023 provided complete data, which were used as the final sample in the current study.

### Measures

Loneliness was assessed using a German adapted version of the six-item De Jong Gierveld Loneliness Scale, which was developed specifically for use in large surveys of older adults [20]. Psychometric studies have validated its reliability and validity [21]. The scale comprises two subscales based on the emotional-social theoretical conceptualization of loneliness: three items measuring emotional loneliness, capturing the absence of intimate relationships and feelings of emptiness and rejection (e.g., “Ich vermisse Leute, bei denen ich mich wohl fühle” [English translation: “I miss having people around me that I feel comfortable with”]; Cronbach’s α = 0.73), and three items measuring social loneliness, capturing the lack of a broader social network and support system (e.g., “Es gibt genug Menschen, die mir helfen würden, wenn ich Probleme habe” [English translation: “There are enough people I can count on in case of a misfortune”]; Cronbach’s α = 0.83). Mean scores were calculated, with scores ranging from 0 (low loneliness) to 3 (high loneliness) after appropriate reverse coding of positively worded items.

Functional limitations were measured using the Global Activity Limitation Indicator (GALI), which assessed whether participants had been limited in their usual activities due to health problems for at least the past 6 months [22, 23]. Response options included three categories: “severely limited”, “limited”, and “not limited”, hereafter referred to as severe, moderate, and none. Additional covariates included age (continuous), gender (male/female), education level based on the International Standard Classification of Education (ISCED) categorized as low (ISCED 0–2), medium (ISCED 3–4), and high (ISCED 5–6), and number of chronic diseases based on a comprehensive health questionnaire covering cardiovascular diseases, diabetes, musculoskeletal conditions, and other health problems, with the total count of reported conditions used as a continuous variable.

### Data analysis

First, descriptive statistics of all variables were calculated using means and frequencies. To examine the relationships between loneliness, functional limitation status and age group, we conducted analysis of variance (ANOVA) with survey weights provided in the dataset to account for the survey design and improve population representativeness. ANOVA was chosen as the primary analytical approach because it is well suited for testing mean differences between groups defined by categorical factors and, importantly, for detecting interaction effects between these factors, both of which are central to our research question. Separate analyses were conducted for emotional loneliness and social loneliness as dependent variables, with age group (40–65 years vs. 66 + years), functional limitation status (no limitations, moderate limitations, severe limitations), and their interaction as independent variables. ANOVA was used to test for main effects (age group, functional limitation status) and interactions (age group * limitation status). Post hoc pairwise comparisons between limitation categories within each age group were conducted using estimated marginal means with Bonferroni correction for multiple testing. Additionally, to examine whether the broad dichotomy of 40–65 versus 66 + years obscured meaningful heterogeneity within the older population, we conducted supplementary analyses using a three-group age categorization (40–65, 66–79, and 80 + years), which allowed us to assess whether patterns of loneliness varied across later-life stages. To assess the robustness of findings, we fitted additional models controlling for gender (similar results emerged for both genders), education level, and number of chronic conditions. All analyses were performed using the R statistical software.

## Results

As shown in Table 1, the study sample comprised 3,984 participants, with 1,426 participants aged 65 years or younger and 2,558 participants aged 66 years or older. The majority of participants (65.3%) reported no functional limitations, while 23.8% reported moderate functional limitations and 10.9% reported severe functional limitations, with slightly higher proportions of functional limitations observed in the older age group. Female participants comprised 52.1% of the overall sample. Educational attainment was predominantly high (50.7%) or medium (46.0%).

**Table 1:** Sample Characteristics

	Overall	<= 65	66+
*N*	3,984	1,426	2,558
Emotional Loneliness (mean (SD))	1.72 (0.61)	1.74 (0.63)	1.71 (0.60)
Social Loneliness (mean (SD))	1.77 (0.60)	1.75 (0.62)	1.78 (0.60)
Functional Limitations (%)			
None	65.3	68.7	63.4
Moderate	23.8	21.6	25.0
Severe	10.9	9.7	11.6
Age (mean (SD))	69.03 (11.07)	57.04 (6.52)	75.71 (6.50)
Gender = Female (%)	52.1	57.3	49.3
Education (%)			
Low	3.4	2.4	3.9
Medium	46.0	47.8	44.9
High	50.7	49.8	51.1
Chronic Conditions (mean (SD))	2.74 (2.01)	2.02 (1.70)	3.14 (2.06)

*N*  sample size, *SD*  Standard Deviation

**Fig. 1:**
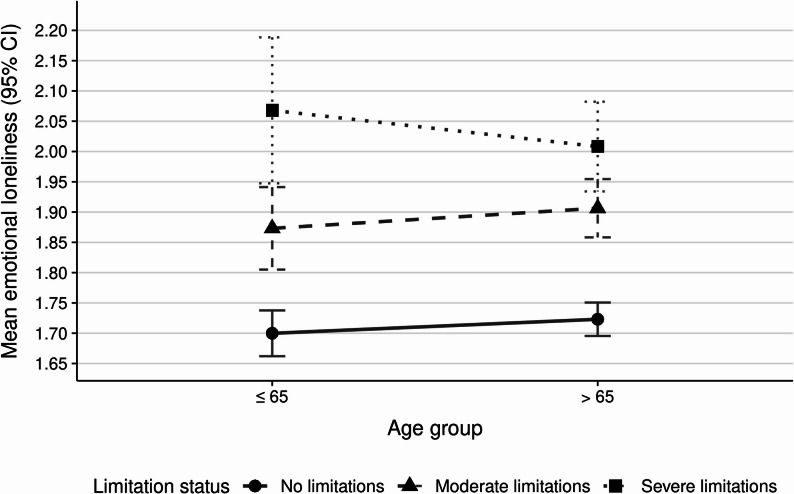
Emotional loneliness by age and limitation status

As depicted in Fig. 1; Table 2, the ANOVA for emotional loneliness revealed a significant main effect of functional limitation status (F(2, 3978) = 52.91, *p* < .001), with more severe functional limitations being associated with higher emotional loneliness, while no significant main effect of age group (F(1, 3978) = 0.00, *p* = .962) or age group by functional limitation status interaction (F(2, 3978) = 1.10, *p* = .334) was observed. Post hoc analyses showed significant differences among all functional limitation categories in the middle-aged group (40–65). In the older group (66+), “No limitations” differed significantly from both “Moderate” and “Severe,” whereas the “Moderate” vs “Severe” comparison was not significant, with participants reporting no functional limitations having lower emotional loneliness scores.

**Table 2:** ANOVA for Emotional Loneliness

Source	SS	df	F	*p*
Age group	0.00	1	0.00	0.962
Limitation status	39.30	2	52.91^***^	< 0.001
Age group × Limitation status	0.80	2	1.10	0.334
Residuals	1479.10	3978		

R² = 0.039, adjusted R² = 0.037. *** *p* < .001

**Fig. 2:**
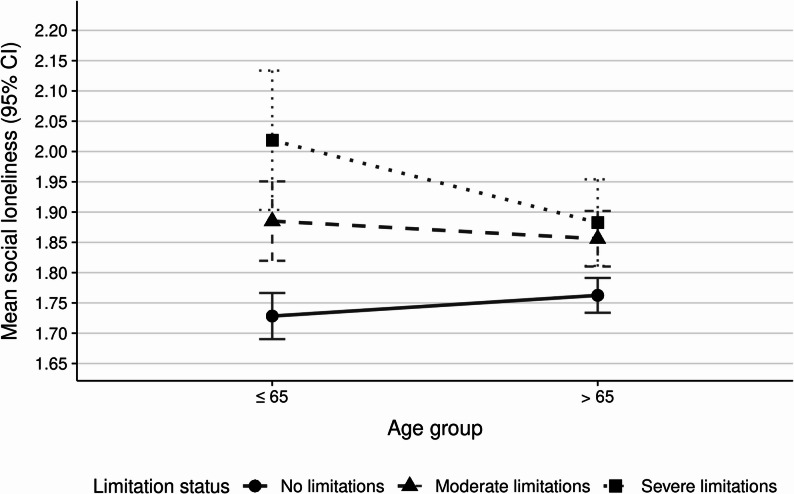
Social loneliness by age and limitation status

As depicted in Fig. 2; Table 3, the ANOVA for social loneliness also revealed a significant main effect of functional limitation status (F(2, 3978) = 35.29, *p* < .001), with more severe functional limitations being associated with higher social loneliness, and a significant age group by functional limitation status interaction (F(2, 3978) = 4.31, *p* = .014). The interaction effect indicated that the relationship between functional limitation status and social loneliness differed between age groups, with stronger social loneliness differences between functional limitation categories being observed in the middle-aged compared to the older age group. Post hoc analyses demonstrated significant differences between all functional limitation categories within the middle-aged group, with participants reporting no functional limitations having lower social loneliness scores compared to those with moderate functional limitations and severe functional limitations, and participants with moderate functional limitations having lower scores than those with severe functional limitations. In the older age group, significant differences were found between no functional limitations and moderate functional limitations and between no functional limitations and severe functional limitations, while the difference between moderate and severe functional limitations was not statistically significant.

**Table 3:** ANOVA for Social Loneliness

Source	SS	df	F	p
Age group	1.20	1	3.31	.069
Limitation status	26.20	2	35.29^***^	< .001
Age group × Limitation status	3.20	2	4.31^*^	.014
Residuals	1477.30	3978		

R² = .020, adjusted R² = .019. * *p* < .05, *** *p* < .001

When controlling for gender, education, and number of chronic conditions, the pattern of results remained largely consistent, though moderate changes in statistical significance were observed (Appendix Table A1 and Table A2). For emotional loneliness, the main effect of functional limitation status remained significant, and a significant main effect of age group emerged. For social loneliness, both the main effect of functional limitation status and the age group by functional limitation status interaction remained significant, with the main effect of age group becoming significant. To examine heterogeneity within the older population, we repeated the analyses using three age groups (40–65, 66–79, and 80 + years). For emotional loneliness, the interaction remained non-significant (F(4, 3975) = 0.99, *p* = .414), with consistent patterns across all three age groups. For social loneliness, the interaction was marginally non-significant (F(4, 3975) = 2.16, *p* = .071), but post hoc comparisons revealed a clear gradient: All pairwise limitation comparisons were significant in the middle-aged group (*p*s < 0.007), two of three were significant in the 66–79 group, and none reached significance in the 80 + group. This reinforces the main finding that functional limitations are most strongly associated with social loneliness among comparatively younger adults, with progressively weaker differentiation at older ages (see Appendix Figures A1 and A2).

## Discussion

We investigated whether people with functional limitations differ from those without functional limitations in their experience of emotional and social loneliness, and whether these differences vary across age groups. Our findings largely support both hypotheses. We found that participants with functional limitations reported significantly higher levels of both emotional and social loneliness compared to those without functional limitations, with progressive increases observed from no functional limitations to moderate functional limitations to severe functional limitations. Importantly, while emotional loneliness showed consistent patterns across age groups, social loneliness displayed a significant interaction with age, where middle-aged participants demonstrated much stronger progressions in social loneliness from no functional limitations to moderate and severe functional limitations compared to older adults. These findings remained robust even when controlling for gender, education, and chronic health conditions, suggesting that individuals with functional limitations experience loneliness to a different degree depending on age group. A sensitivity analysis using a more differentiated three-group age categorization (40–65, 66–79, and 80 + years) further confirmed these findings. For emotional loneliness, the pattern was consistent across all three age groups. For social loneliness, the gradient by limitation status was strongest among middle-aged adults and progressively weaker at older ages, with no significant limitation-based differences in the 80 + group. These results reinforce the conclusion that functional limitations are associated with particularly pronounced social loneliness implications in midlife.

These results both confirm and contradict previous studies examining loneliness among individuals with functional limitations. Our findings confirm previous research demonstrating that individuals with functional limitations experience elevated levels of loneliness compared to those without limitations [15, 18]. At the same time, our findings warrant a more nuanced interpretation in relation to the disability paradox [16, 17]. The paradox has primarily been described for broad evaluations of quality of life and subjective well-being rather than for every specific psychosocial outcome. Our results do not necessarily contradict this broader pattern; they suggest instead that the adaptive processes underlying the paradox may not extend uniformly to all domains, and that loneliness remains elevated among individuals with functional limitations even where global well-being may be preserved. This study extends beyond previous research that has primarily focused on establishing the presence of differences in loneliness between people with and without functional limitations by demonstrating that the extent of these differences depends on both the type of loneliness examined and the age group considered. While earlier disability studies treated loneliness mostly as a unidimensional construct, our findings reveal that functional limitations have differential impacts on emotional versus social loneliness, with particularly pronounced age-related variations in social loneliness that were not as apparent for emotional loneliness. This nuanced understanding suggests that the relationship between functional limitations and loneliness is more complex than previously recognized, highlighting the importance of considering multiple dimensions of loneliness and sociodemographic factors when examining the psychosocial health of people with functional limitations.

### Explanations

Several possible interpretations may help contextualize the observed differences, though it should be noted that the mechanisms discussed below are not directly measured in our data and should therefore be understood as hypotheses for future research rather than as conclusions substantiated by the present analyses. The heightened loneliness among people with functional limitations likely reflects multiple barriers to social participation and social exclusion [24, 25].

One possible interpretation is that the particularly strong association between functional limitations and social loneliness in middle-aged adults may stem from age-specific psycho-social expectations and life-stage demands that are more difficult to navigate with functional constraints. Middle-aged adults typically face greater pressure to establish or maintain careers and build or maintain romantic relationships during a period when social participation is heavily emphasized and expected [26].

Drawing on Festinger’s social comparison theory, it is conceivable that middle-aged adults with functional limitations face particularly distressing upward comparisons with their highly active, socially engaged peers, though this mechanism was not directly assessed in our study [27]. In contrast, older adults might compare themselves more to age-matched peers who are also experiencing normative declines in social activity, making these comparisons less problematic. This discrepancy between self and comparison others might intensify feelings of loneliness. Thus, the same functional limitation that severely impacts social well-being in midlife may have diminished effects among older adults.

If this interpretation holds, functional limitations may create more pronounced barriers to meeting these developmental milestones and social role expectations compared to older adults. Furthermore, middle-aged adults with functional limitations may experience greater relative disadvantage in social contexts that are designed for more functional individuals, while older adults may benefit from environments and services that are more accommodating to functional limitations [28].

More broadly, the interaction between functional limitations and age-related changes in loneliness may be understood through a convergence mechanism. As individuals age, normative increases in loneliness occur due to bereavement, retirement, and declining health, meaning that older adults without functional limitations already experience elevated baseline levels of loneliness. Consequently, the additional burden imposed by functional limitations may become less distinguishable from this age-normative loneliness trajectory in later life. In contrast, middle-aged adults typically maintain more active social lives, and functional limitations may represent a more disruptive deviation from their expected social trajectory, producing the starker differences in social loneliness observed in our data. However, this hypothesis should be investigated by future studies.

### Implications

The findings of this study have important theoretical and practical implications for understanding and addressing loneliness among individuals with functional limitations. Theoretically, our results challenge unidimensional conceptualizations of loneliness in disability research and highlight the necessity of examining different types of loneliness separately, as they exhibit distinct patterns across age groups [15]. The differential age-related effects observed for social versus emotional loneliness suggest that theoretical models of disability and psychosocial well-being must account for sociodemographic factors.

From a practical standpoint, our findings identify middle-aged adults with functional limitations as a particularly vulnerable group, warranting targeted interventions tailored to their specific challenges. This vulnerability is noteworthy in light of recent evidence suggesting that functional limitations are increasing among newer cohorts in Europe, with studies demonstrating higher levels of functional limitations in cohorts born after 1960 compared to earlier generations [29, 30]. This trend raises the possibility that a growing population of middle-aged adults with limitations could be at heightened risk for social loneliness and its associated negative health consequences, though longitudinal research would be needed to confirm this. These findings suggest that age-specific intervention strategies addressing the unique challenges faced by comparatively younger adults with functional limitations may be warranted.

### Limitations

This study has several limitations that should be acknowledged. First, our sample was limited to German participants, which may restrict the generalizability of findings to other cultural and national contexts [7]; however, this focus allowed us to examine a large, population-based sample. In a similar vein, future studies with larger samples could explore even more fine-grained age categorizations to further elucidate how the disability-loneliness association unfolds across later-life stages. Going even further, future research could employ non-linear regression frameworks in which age is treated as a continuous variable and interacted with functional limitation status, which would allow for a more fine-grained assessment of potential nonlinearities in the association between functional limitations and loneliness across the age range. Second, we utilized a relatively brief loneliness instrument rather than more comprehensive measures; nevertheless, this approach enabled us to differentiate between emotional and social loneliness dimensions, which has been identified as a critical gap in previous disability and loneliness research [20]. While the six-item De Jong Gierveld Scale has been validated for large-scale survey research and enabled the crucial distinction between emotional and social loneliness, future studies could still benefit from employing longer and more comprehensive loneliness instruments that offer greater measurement precision and a wider score range, potentially revealing more subtle differences between groups. Furthermore, the possible interpretations discussed above represent plausible but empirically untested mechanisms. Future research using longitudinal designs and direct measures of these mediating processes would be needed to substantiate these hypotheses. Finally, the cross-sectional design of our study prevents causal inferences about the relationship between functional limitations and loneliness. Longitudinal studies, such as those by Abaei and Martin employing cross-lagged panel models to examine the dynamic interplay between loneliness and cognition in older adults [13, 14], illustrate the kind of designs that would be needed to establish temporal precedence and causal pathways in this domain. In a similar vein, future studies could particularly benefit from adopting longitudinal designs by clarifying the temporal dynamics between aging, functional limitations, and loneliness. Such designs would allow researchers to disentangle whether functional limitations lead to increased loneliness or whether loneliness itself accelerates functional decline, and how these processes may differ across the life course.

## Conclusions

Functional limitations are consistently associated with increased loneliness, but the magnitude of these associations varies significantly depending on the type of loneliness and age group considered. The particularly pronounced social loneliness differences among middle-aged adults with functional limitations highlight this population as especially vulnerable and deserving of targeted attention. These results underscore the importance of adopting nuanced approaches that consider the multifaceted nature of psychosocial health among people with functional limitations.

## Supplementary Information

Supplementary Material 1.

## Data Availability

The datasets analysed during the current study are publicly available from the Research Data Centre of the German Centre of Gerontology (DZA). Data can be accessed at https://www.dza.de/en/research/fdz/german-ageing-survey/data-access.

## References

[1] Hawkley LC, Cacioppo JT. Loneliness Matters: A Theoretical and Empirical Review of Consequences and Mechanisms. ann behav med. 2010;40:218–27. 10.1007/s12160-010-9210-8. 10.1007/s12160-010-9210-8 PMC3874845 20652462

[2] Weiss RS, Loneliness. The experience of emotional and social isolation. Cambridge, MA, US: The MIT Press; 1973.

[3] Beller J, Wagner A, Disentangling, Loneliness. Differential Effects of Subjective Loneliness, Network Quality, Network Size, and Living Alone on Physical, Mental, and Cognitive Health. J Aging Health. 2018;30:521–39. 10.1177/0898264316685843. 28553795 10.1177/0898264316685843

[4] Holt-Lunstad J, Smith TB, Baker M, Harris T, Stephenson D. Loneliness and Social Isolation as Risk Factors for Mortality: A Meta-Analytic Review. Perspect Psychol Sci. 2015;10:227–37. 10.1177/1745691614568352. 25910392 10.1177/1745691614568352

[5] National Academies of Sciences, Engineering, and, Medicine, U.S. National Academies of Sciences, Engineering, and Medicine (U.S.), National Academies of Sciences, Engineering, and Medicine (U.S.), National Academies of Sciences, Engineering, and Medicine (U.S.), National Academies of Sciences, Engineering, and Medicine (U.S.), editors. Social isolation and loneliness in older adults: opportunities for the health care system. Washington, DC: the National Academies Press; 2020. 32510896

[6] Holt-Lunstad J. The Potential Public Health Relevance of Social Isolation and Loneliness: Prevalence, Epidemiology, and Risk Factors. Public Policy Aging Rep. 2017;27:127–30. 10.1093/ppar/prx030.

[7] Beller J, Wagner A. Loneliness and Health: The Moderating Effect of Cross-Cultural Individualism/Collectivism. J Aging Health. 2020;089826432094333. 10.1177/0898264320943336. 10.1177/0898264320943336 32723203

[8] Cacioppo JT, Hawkley LC, Crawford LE, Ernst JM, Burleson MH, Kowalewski RB, et al. Loneliness Health: Potential Mechanisms: Psychosom Med. 2002;64:407–17. 10.1097/00006842-200205000-00005. 12021415 10.1097/00006842-200205000-00005

[9] Luhmann M, Hawkley LC. Age differences in loneliness from late adolescence to oldest old age. Dev Psychol. 2016;52:943–59. 10.1037/dev0000117. 27148782 10.1037/dev0000117 PMC8015413

[10] Yang K, Victor C. Age and loneliness in 25 European nations. Aging Soc. 2011;31:1368–88. 10.1017/S0144686X1000139X.

[11] Qualter P, Hennessey A, Yang K, Chester KL, Klemera E, Brooks F. Prevalence and Social Inequality in Youth Loneliness in the UK. IJERPH. 2021;18:10420. 10.3390/ijerph181910420. 34639720 10.3390/ijerph181910420 PMC8507796

[12] Wrzus C, Hänel M, Wagner J, Neyer FJ. Social network changes and life events across the life span: a meta-analysis. Psychol Bull. 2013;139:53–80. 10.1037/a0028601. 22642230 10.1037/a0028601

[13] Abaei E, Martin P. Loneliness and cognitive function in older. adults: a longitudinal analysis. Innov Aging. 2024;8Supplement_1:894. 10.1093/geroni/igae098.2889.

[14] Abaei E, Martin P. Unraveling the dynamics of loneliness and cognition in late life: a cross-lagged panel model. Front Public Health. 2024;12. 10.3389/fpubh.2024.1425403. 10.3389/fpubh.2024.1425403 PMC11335492 39171310

[15] Gómez-Zúñiga B, Pousada M, Armayones M. Loneliness and disability: A systematic review of loneliness conceptualization and intervention strategies. Front Psychol. 2023;13. 10.3389/fpsyg.2022.1040651. 10.3389/fpsyg.2022.1040651 PMC9905422 36760915

[16] Albrecht GL, Devlieger PJ. The disability paradox: high quality of life against all odds. Soc Sci Med. 1999;48:977–88. 10.1016/S0277-9536(98)00411-0. 10390038 10.1016/s0277-9536(98)00411-0

[17] van Loon AM, Depla MFIA, Hertogh CMPM, Huisman M, Kok AAL. The Disability Paradox? Trajectories of Well-Being in Older Adults With Functional Decline. J Aging Health. 2023;35:125–37. 10.1177/08982643221108660. 35713401 10.1177/08982643221108660 PMC9755699

[18] Bishop GM, Llewellyn G, Kavanagh AM, Badland H, Bailie J, Stancliffe R, et al. Disability-related inequalities in the prevalence of loneliness across the lifespan: trends from Australia, 2003 to 2020. BMC Public Health. 2024;24:621. 10.1186/s12889-024-17936-w. 38413942 10.1186/s12889-024-17936-w PMC10898179

[19] Klaus D, Engstler H, Mahne K, Wolff JK, Simonson J, Wurm S et al. Cohort Profile: The German Ageing Survey (DEAS). International Journal of Epidemiology. 2017;46:1105–1105g. 10.1093/ije/dyw326. 10.1093/ije/dyw326 PMC5837219 28180273

[20] Gierveld JDJ, Tilburg TV. A 6-Item Scale for Overall, Emotional, and Social Loneliness: Confirmatory Tests on Survey Data. Res Aging. 2006;28:582–98. 10.1177/0164027506289723.

[21] De Jong Gierveld J, Van Tilburg T. The De Jong Gierveld short scales for emotional and social loneliness: tested on data from 7 countries in the UN generations and gender surveys. Eur J Ageing. 2010;7:121–30. 10.1007/s10433-010-0144-6. 20730083 10.1007/s10433-010-0144-6 PMC2921057

[22] Van Oyen H, Bogaert P, Yokota RTC, Berger N. Measuring disability: a systematic review of the validity and reliability of the Global Activity Limitations Indicator (GALI). Archives Public Health. 2018;76. 10.1186/s13690-018-0270-8. 10.1186/s13690-018-0270-8 PMC5985596 29881544

[23] van Oyen H, Van der Heyden J, Perenboom R, Jagger C. Monitoring population disability: evaluation of a new Global Activity Limitation Indicator (GALI). Sozial- und Präventivmedizin. 2006;51:153–61. 10.1007/s00038-006-0035-y. 17191540 10.1007/s00038-006-0035-y

[24] Bezyak JL, Sabella S, Hammel J, McDonald K, Jones RA, Barton D. Community participation and public transportation barriers experienced by people with disabilities. Disabil Rehabil. 2020;42:3275–83. 10.1080/09638288.2019.1590469. 30991852 10.1080/09638288.2019.1590469

[25] Jespersen LN, Michelsen SI, Tjørnhøj-Thomsen T, Svensson MK, Holstein BE, Due P. Living with a disability: a qualitative study of associations between social relations, social participation and quality of life. Disabil Rehabil. 2019;41:1275–86. 10.1080/09638288.2018.1424949. 29357697 10.1080/09638288.2018.1424949

[26] Infurna FJ, Gerstorf D, Lachman ME. Midlife in the 2020s: Opportunities and challenges. Am Psychol. 2020;75:470–85. 10.1037/amp0000591. 32378943 10.1037/amp0000591 PMC7347230

[27] Festinger LA, Theory of Social Comparison Processes. Hum Relat. 1954;7:117–40. 10.1177/001872675400700202.

[28] Wiles JL, Leibing A, Guberman N, Reeve J, Allen RES. The Meaning of Aging in Place to Older People. Gerontologist. 2012;52:357–66. 10.1093/geront/gnr098. 21983126 10.1093/geront/gnr098

[29] Beller J, Epping J. Disability trends in Europe by age-period-cohort analysis: Increasing disability in younger cohorts. Disabil Health J. 2020;100948. 10.1016/j.dhjo.2020.100948. 10.1016/j.dhjo.2020.100948 32690322

[30] Beller J, Luy M, Giarelli G, Regidor E, Lostao L, Tetzlaff J, et al. Trends in Activity Limitations From an International Perspective: Differential Changes Between Age Groups Across 30 Countries. J Aging Health. 2022;089826432211411. 10.1177/08982643221141123. 10.1177/08982643221141123 PMC10302378 36426682

