# Supplementary material for: Functional limitations and loneliness in middle-aged and older adults: differentiating emotional loneliness and social loneliness
Source: BMC Public Health. 2026 Jun 1;26:1758. doi: 10.1186/s12889-026-27997-8 (PMC13224435; doi:10.1186/s12889-026-27997-8)

APPENDIX

*Table A1. ANOVA for Emotional Loneliness (With Control Variables)*

| *Source* | *SS* | *df* | *F* | *p* |
| --- | --- | --- | --- | --- |
| Age group | 5.67 | 1 | 16.11^***^ | < .001 |
| Limitation status | 15.10 | 2 | 21.46^***^ | < .001 |
| Gender | 1.78 | 1 | 5.06^*^ | .024 |
| Education | 15.93 | 2 | 22.65^***^ | < .001 |
| Number of chronic conditions | 60.01 | 1 | 170.63^***^ | < .001 |
| Age group × Limitation status | 0.84 | 2 | 1.19 | .303 |
| Residuals | 1397.63 | 3974 |  |  |

*Note. R² = .092, adjusted R² = .089. * p < .05, *** p < .001*

*Table A2. ANOVA for Social Loneliness (With Control Variables)*

| *Source* | *SS* | *df* | *F* | *p* |
| --- | --- | --- | --- | --- |
| Age group | 6.16 | 1 | 17.49^***^ | < .001 |
| Limitation status | 15.79 | 2 | 22.41^***^ | < .001 |
| Gender | 47.60 | 1 | 135.12^***^ | < .001 |
| Education | 0.26 | 2 | 0.37 | .694 |
| Number of chronic conditions | 28.92 | 1 | 82.09^***^ | < .001 |
| Age group × Limitation status | 3.81 | 2 | 5.41^**^ | .004 |
| Residuals | 1399.97 | 3974 |  |  |

*Note. R² = .071, adjusted R² = .069. ** p < .01, *** p < .001*

**Figure A1.** Emotional loneliness by 3 age groups and limitation status


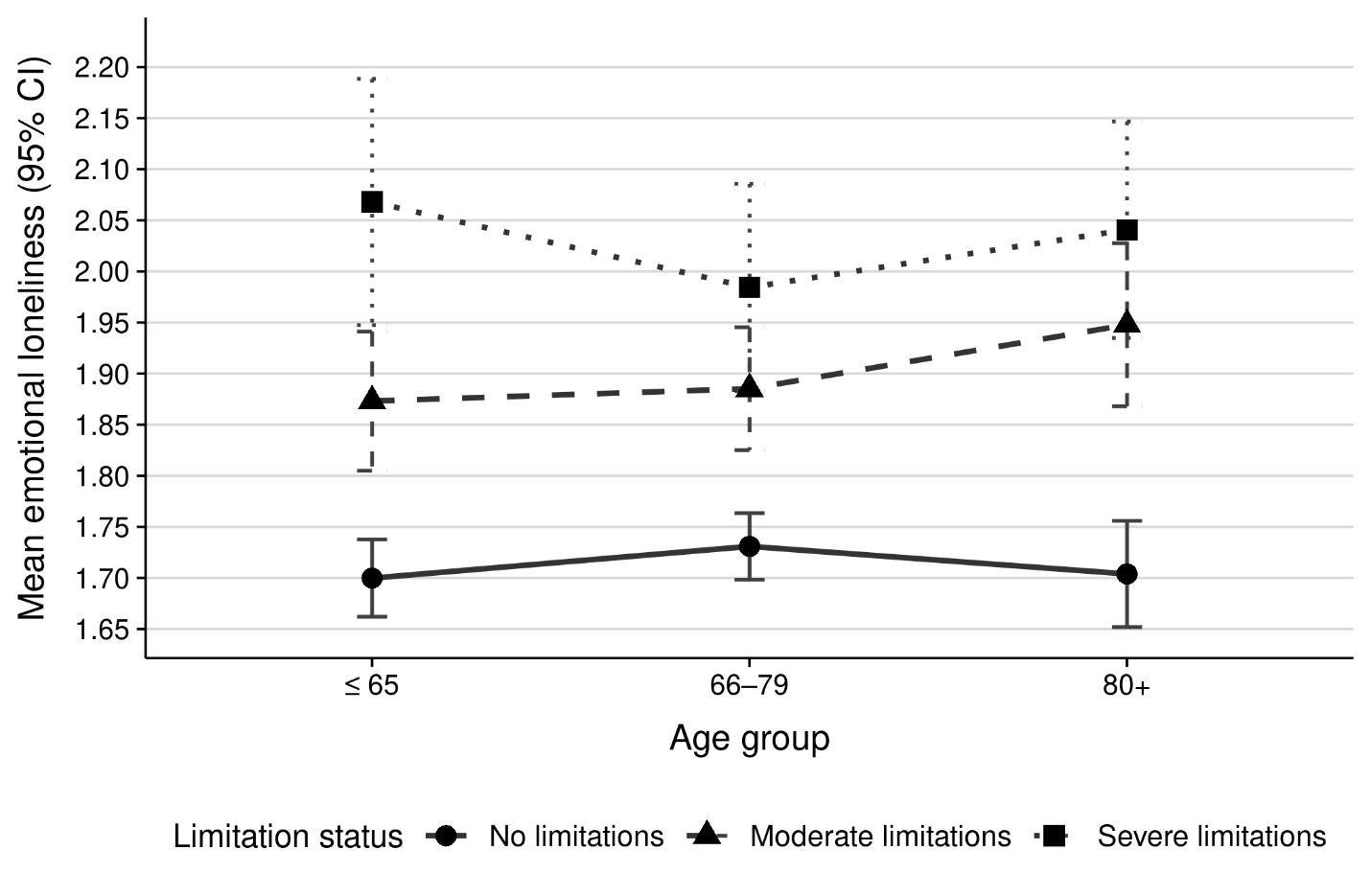


**Figure A2.** Social loneliness by 3 age groups and limitation status


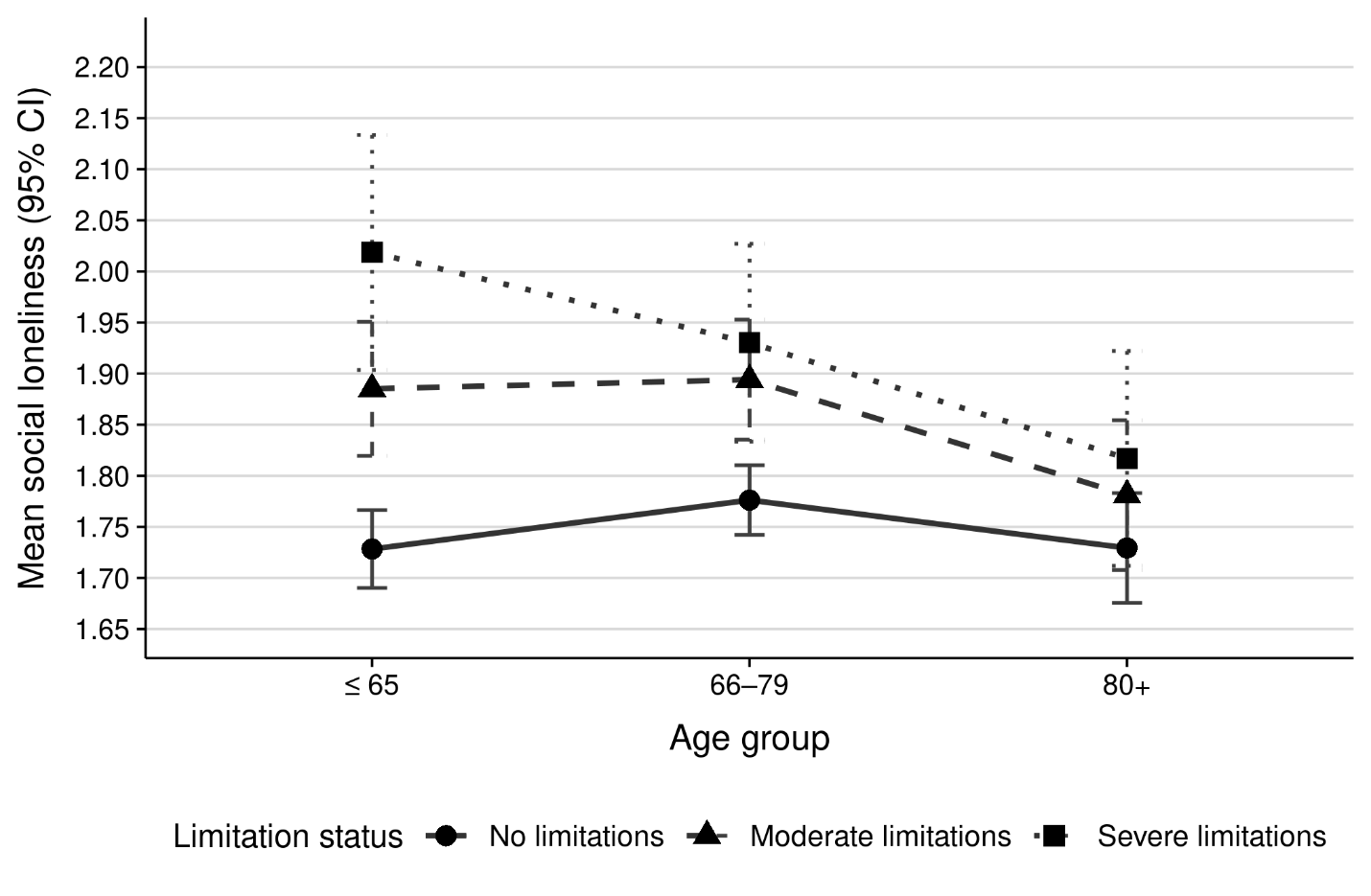

Supplement: Supplementary file 1 — Supplementary Material 1. [file 12889_2026_27997_MOESM1_ESM.docx]
